# Infliximab therapy together with tyrosine kinase inhibition targets leukemic stem cells in chronic myeloid leukemia

**DOI:** 10.1186/s12885-019-5871-2

**Published:** 2019-07-04

**Authors:** Oliver Herrmann, Maja Kim Kuepper, Marlena Bütow, Ivan G. Costa, Iris Appelmann, Fabian Beier, Tom Luedde, Till Braunschweig, Steffen Koschmieder, Tim H. Brümmendorf, Mirle Schemionek

**Affiliations:** 1 Department of Hematology Oncology Hemostaseology and Stem Cell Transplantation Faculty of Medicine, University Hospital RWTH Aachen, Pauwelsstr 30, 52074 Aachen, Germany; 2 IZKF Research Group Bioinformatics Institute for Biomedical Engineering, RWTH Aachen University, Aachen, Germany; 3 Department of Medicine III, University Hospital RWTH Aachen, Aachen, Germany; 4 Department of Pathology, University Hospital RWTH Aachen, Aachen, Germany

**Keywords:** CML, Leukemic stem cells, Inflammation, Tyrosine kinase inhibitor, Infliximab, TNF, Therapy, Mouse model

## Abstract

**Background:**

Expression of Bcr-Abl in hematopoietic stem cells is sufficient to cause chronic myeloid leukemia (CML) and tyrosine kinase inhibitors (TKI) induce molecular remission in the majority of CML patients. However, the disease driving stem cell population is not fully targeted by TKI therapy, and leukemic stem cells (LSC) capable of re-inducing the disease can persist. Single-cell RNA-sequencing technology recently identified an enriched inflammatory gene signature with TNFα and TGFβ being activated in TKI persisting quiescent LSC. Here, we studied the effects of human TNFα antibody infliximab (IFX), which has been shown to induce anti-inflammatory effects in mice, combined with TKI treatment on LSC function.

**Methods:**

We first performed GSEA-pathway analysis using our microarray data of murine LSK cells (lin^−^; Sca-1^+^; c-kit^+^) from the SCLtTA/Bcr-Abl CML transgenic mouse model. Bcr-Abl positive cell lines were generated by retroviral transduction. Clonogenic potential was assessed by CFU (colony forming unit). CML mice were treated with nilotinib or nilotinib plus infliximab, and serial transplantation experiments were performed.

**Results:**

Likewise to human CML, TNFα signaling was specifically active in murine CML stem cells, and ectopic expression of Bcr-Abl in murine and human progenitor cell lines induced TNFα expression. In vitro exposure to human (IFX) or murine (MP6-XT22) TNFα antibody reduced clonogenic growth of CML cells. Interestingly, TNFα antibody treatment enhanced TKI-induced effects on immature cells in vitro. Additionally, in transplant and serial transplant experiments, using our transgenic CML mouse model, we could subsequently show that IFX therapy boosted TKI-induced effects and further reduced the proportion of malignant stem cells in vivo.

**Conclusion:**

TNFα signaling is induced in CML stem cells, and anti-inflammatory therapy enhances TKI-induced decline of LSC, confirming that successful targeting of persisting CML stem cells can be enhanced by addressing their malignant microenvironment simultaneously.

**Electronic supplementary material:**

The online version of this article (10.1186/s12885-019-5871-2) contains supplementary material, which is available to authorized users.

## Background

Chronic myeloid leukemia (CML) is a myeloproliferative neoplasm developing upon acquisition of the reciprocal translocation t (9;22) within the hematopoietic stem cell (HSC) compartment. The mutation gives rise to the constitutively activated tyrosine kinase Bcr-Abl that contains multiple interaction sites, activating a variety of signaling pathways. Bcr-Abl positive leukemic cells show increased proliferation, differentiation, genomic instability and survival [1, 2]. The implementation of tyrosine kinase inhibitors (TKI) induced very high response rates but while the majority of newly diagnosed CML-CP patients respond well to TKI treatment, about one-third develop primary or secondary resistance or intolerance to TKIs. Beyond that, others and we have previously demonstrated that even in patients responding well to TKI therapy the disease-driving CML stem cell population (leukemic stem cells, LSCs) persists [3–5]. This LSC persistence has been assigned to a lack of oncogene addiction within the malignant stem cell compartment. As a result, treatment-free remission can currently be achieved only in about 12% of patients upon first-line imatinib treatment [6].

Recent data have shown that therapy persistence in LSCs is at least in part mediated via the stem cell specific microenvironment, and an increasing number of reports suggest that the BM niche composition in CML differs from a normal niche. Soluble factors that are abnormally produced in CML include IL-1 α, IL-1 β, IL-6, TNFα, MIP-1α MIP-1β, G-CSF or CXCL12 [7–9]. Some of them, such as IL-1 and IL-6, have already been shown to support malignant stem cell function in CML [10–13].

Interestingly, many of these cytokines can be induced by TNFα, and TNFα is likewise increased in CML patients as well as in a transgenic SCLtTA/Bcr-Abl CML mouse model [8, 9]. Although LSCs are difficult to separate from their normal counterparts due to a similar immunophenotype and biology, a recent report using single-cell RNA-sequencing technology achieved to discriminate Bcr-Abl positive and negative stem cells via expression of the oncogene itself. Using this approach, the authors identified a malignant stem cell population reflecting a gene signature associated with either cycling or quiescence, with the latter population persisting in patients despite therapy. Signaling pathway analysis revealed activated TGFβ and TNFα signaling via NF-kB in these persisting LSCs [14].

Infliximab (IFX) is a chimeric antibody neutralizing TNFα in humans and is approved for multiple applications including ulcerative colitis, rheumatoid arthritis, Crohn’s disease, or psoriatic arthritis. In a variety of preclinical mouse models, IFX application has been used as an anti-inflammatory therapy showing reduction of TNFα in the mice upon application [15–22]. Recently, it has been shown that reduction of TNFα in mice along with a decline of further pro-inflammatory cytokines is not mediated via a direct interaction of IFX and TNFα and the mechanism underlying the anti-inflammatory response in mice has thus to be clarified [23].

Similar to human CML, we here show that TNFα signaling is activated in murine CML stem cells and that TNFα targeting enhanced TKI-induced reduction of clonogenic activity. Aiming to test an antibody-based therapeutic approach, targeting inflammation along with TKI therapy in vivo, we subsequently applied our transgenic SCLtTA/Bcr-Abl CML mouse model [24]. In this model, malignant stem cells were further reduced by IFX therapy combined with TKI as compared to TKI standard treatment alone.

## Methods

### Cell culture

32Dcl3 (here after named as 32D) and BA/F3 cells (ACC-411, ACC-300, DSMZ, 2018–01) were cultured as described previously [25, 26]. TF-1 cells (ACC-334, DSMZ, 2018–01) were cultured using RPMI 1640/10%FCS/GM-CSF (5 ng/ml). All cell lines were routinely tested for mycoplasma using PCR. Authentication of cell lines was performed using qRT-PCR for murine or human housekeeping gene as well as cell surface expression of characteristic receptor expression pattern (CD34, CD11b, Gr-1) using FACS analysis. Primary murine cells were cultured in serum-free BIT9500 cell culture medium (Stem Cell Technologies, Vancouver, BC, Canada) supplemented with mIL-3 (10 ng/ml), mIL-6 (5 ng/ml) and mSCF (50 ng/ml). All cytokines were purchased form ImmunoTools, (Friesoythe, Germany). Further, lineage negative transgenic SCLtTA/Bcr-Abl BM cells were retrovirally infected using MSCV-ER-Hoxb8-Neo plasmid as described previously [27]. ER-HoxB8 derived immortalized progenitor cells were cultured in IMDM containing 10% FBS, 5% SCF-supernatant and 1% Pen-Strep and selected with G418 (1 mg/ml) for 1 week. FACS analysis for Gr-1, CD11b, B220, CD3 and Ter119 (BioLegend, Fell, Germany) were performed demonstrated the absence of mature cell surface markers.

### Isolation of primary cells

Mice were sacrificed by cervical dislocation in isoflurane anesthesia. Murine Bone marrow (BM) cells were isolated from tibia and femora of SCLtTA/Bcr-Abl mice by flushing the marrow with PBS supplemented with 2% fetal calf serum (FCS). Cells were subjected to red blood cell lysis using ammonium-chloride-potassium buffer (0.15 M NH_4_Cl, 1 mM KHCO_3_, 0.1 mM Na_2_-EDTA, pH 7.3). Lineage negative cell isolation was performed by magnetic-activated cell sorting (MACS) using the mouse lineage depletion kit (Milteny Biotec, Bergisch Gladbach, Germany).

### Retroviral transduction

Retroviral transduction was performed following previously described protocols [28, 29]. Briefly, Plate-E packaging cells were transfected using MSCV-BcrAbl-IRES-RFP and MSCV-IRES-RFP empty vector. Viral supernatant was collected after 24 h and subsequently centrifuged onto RetroNectin-coated (Takara Bio Europe/Clontech, France) six-well plates. 1 × 10^6^ 32D and BA/F3 cells were added and cultured for 2 days before FACS sorting for vector encoded RFP expression.

### Real-time quantitative reverse transcriptase–PCR (qRT-PCR)

Total RNA was isolated using Trizol reagent (Thermo Fischer Scientific, Waltham, MA, USA) as described previously [30]. mRNA expression of human and murine TNFα was measured with a 7500 Fast Real-Time PCR cycler (Applied Biosystems, Waltham, MA, USA) using SYBR-Green reagent (Thermo Fischer Scientific) with the following primer pairs: TNF-α forward 5′-GTAGCCCACGTCGTAGCAAA-3′; TNF-α reverse 5′-ACAAGGTACAACCCATCGGC-3′; INFγ forward 5′-ACGGCACAGTCATTGAAAGC-3′; INFγ reverse 5′-TCACCATCCTTTTGCCAGTTC-3′; GAPDH forward 5′-TTGTGCAGTGCCAGCCTC-3′ and GAPDH reverse 5′-CCAATACGGCCAATCCG-3′. Bcr-Abl expression was assessed using hydrolyzing TaqMan probes and primers: Bcr-Abl forward 5′-CGTCAACTCAGCCACTGG-3′; Bcr-Abl reverse 5′-GGCTTCACTCAGACCCTGA-3′; Bcr-Abl probe 5′-FAM-AGCGGCCAGTATCATCTGACTTTGAGC-TAMRA-3′; A20 forward 5′-GAACAGCGATCAGGCCAGG-3′; A20 reverse 5′-GGACAGTTGGGTGTCTCACATT-3′. GAPDH forward and GAPDH reverse primer were used as mentioned above and combined with GAPDH probe 5′-FAM-TCCCGTAGACAAAATGGTGAAGGTCGGT-TAMRA-3′.

### Apoptosis and proliferation assays

For apoptosis and proliferation analyses 5 × 10^5 cells per ml were treated with or without 0.05 ng/ml TNF, 100 nM Nilotinib, 2.5 μg/ml MP6-XT22 or DMSO as control. For proliferation analysis, the cells were stained using Trypan Blue and counted using a hemocytometer after 24 and 48 h of treatment. Early and late apoptotic cells were stained using an APC Annexin V Apoptosis Detection Kit with 7-AAD (BioLegend) upon 48 h of treatment. Proportional distribution was assessed via Flow cytometry analysis using a Gallios flow cytometer (Beckman Coulter, Krefeld, Germany) and Kaluza (Version 1.3) analysis software.

### Preparation of cell lysates, SDS–PAGE and immunoblotting

Cell lysates and western blot (WB) analysis was performed as previously described [29] using following antibodies: pSTAT5^Y694^, STAT5, pIκBα^S32/36^, IκBα (Cell Signaling, Danvers, MA, USA), and GAPDH (Santa Cruz, Heidelberg, Germany).

### Mice and genotyping

CD45.1^+^ SCLtTA (BDF-FVB/N background backcrossed to FVB/N for 4–6 generations) and Bcr-Abl (FVB/N background) mice were genotyped as described previously [24]. Recipient FVB/N CD45.2^+^ mice were bred in-house.

### Colony formation assay

Lineage depleted SCLtTA/Bcr-Abl BM cells were treated for 72 h with TKI 100 nM nilotinib (LC Laboratories, Woburn, MA, USA), 500 μg/ml infliximab (Remicade, Jansen Biologics) or in combination. Nilotinib was added freshly every day. Treated cells were subjected to methylcellulose (MethoCult GF M3434; Stem Cell Technologies). Additionally, murine anti-TNFα antibody (2 μg/ml, clone MP6-XT22, eBioscience, San Diego, CA, USA) was used. Colony numbers were determined on day 7 using a light microscope.

### Bone marrow transplantation and treatment of mice

BM cells were isolated from SCLtTA/Bcr-Abl and wildtype (wt) mice in an FVB/N background. BM cells from three SCLtTA/Bcr-Abl mice were pooled. Transplantation was performed using 1.5 × 10^6^ wt or Bcr-Abl BM cells expressing CD45.1 by tail vain injection. FVB/N 45.2^+^ wt recipients were irradiated using 10 Gy. Mice were treated with cotrimoxazole (Ratiopharm, Ulm, Germany) for 2 weeks after transplantation. Cells were allowed to engraft and expand for 14 days. Bcr-Abl transplanted mice were treated with TKI nilotinib (50 mg/kg, daily) by oral gavage alone or combined therapy with the chimeric antibody infliximab (10 mg/kg, weekly i.v., tail vein). Control mice were treated with vehicle alone or together with human IgG control (10 mg/kg, weekly i.v., Sigma Aldrich, St. Louis, MO, USA). All mice were sacrificed after 2–5 weeks of treatment.

### Flow cytometry analysis

BM cells were isolated from tibie and femora by flushing with PBS/2% FBS. Peripheral blood (PB) was drawn from the orbital plexus. Spleen cells were separated by a 100 μM cell strainer (Greiner Bio-one, Frickenhausen, Germany). Red blood cell lysis was applied using ammonium-chloride-potassium buffer. The following antibodies were used for phenotyping by FACS: CD45.1, CD45.2, Gr-1, CD11b, c-kit, B220 (BioLegend). The LSK cell compartment was analyzed using tricolor- or PE-Cy5 labeled CD4, CD8a, B220 (life technologies, Carlsbad, CA, USA), Gr-1, Ter119 and CD11b (BioLegend) to label lin^+^ cells. Furthermore, lineage-negative cells were analyzed for LSK^+^ cells using c-kit APC-Cy7 (BioLegend) and Sca-1 (biotin labeled first antibody and streptavidin PE-Cy7 secondary antibody, BD Bioscience, Franklin Lakes, NJ, USA). CD45.1-FITC, CD45.2-PE (BioLegend) was used to discriminate between donor and recipient cells. FACS measurements were performed using a Gallios Flow Cytometer (Beckman Coulter, Krefeld, Germany). FACS data were analyzed with Kaluza (Version 1.3) or FlowJo Software (Version 10).

### Statistical analysis

Two-sided Student’s t-test, 1-way or 2-way Anova using Bonferroni post-test (GraphPad Prism software) were used as applicable for statistical analysis. Error bars are given as standard error of the mean (s.e.m). Log-rank test was performed for Kaplan-Meier survival analysis. *p* < 0.05 (*), *p* < 0.01 (**), *p* < 0.001 (***) were considered as statistical significant.

## Results

### TNFα gene expression signature in leukemic stem and progenitor cells

**Fig. 1:**
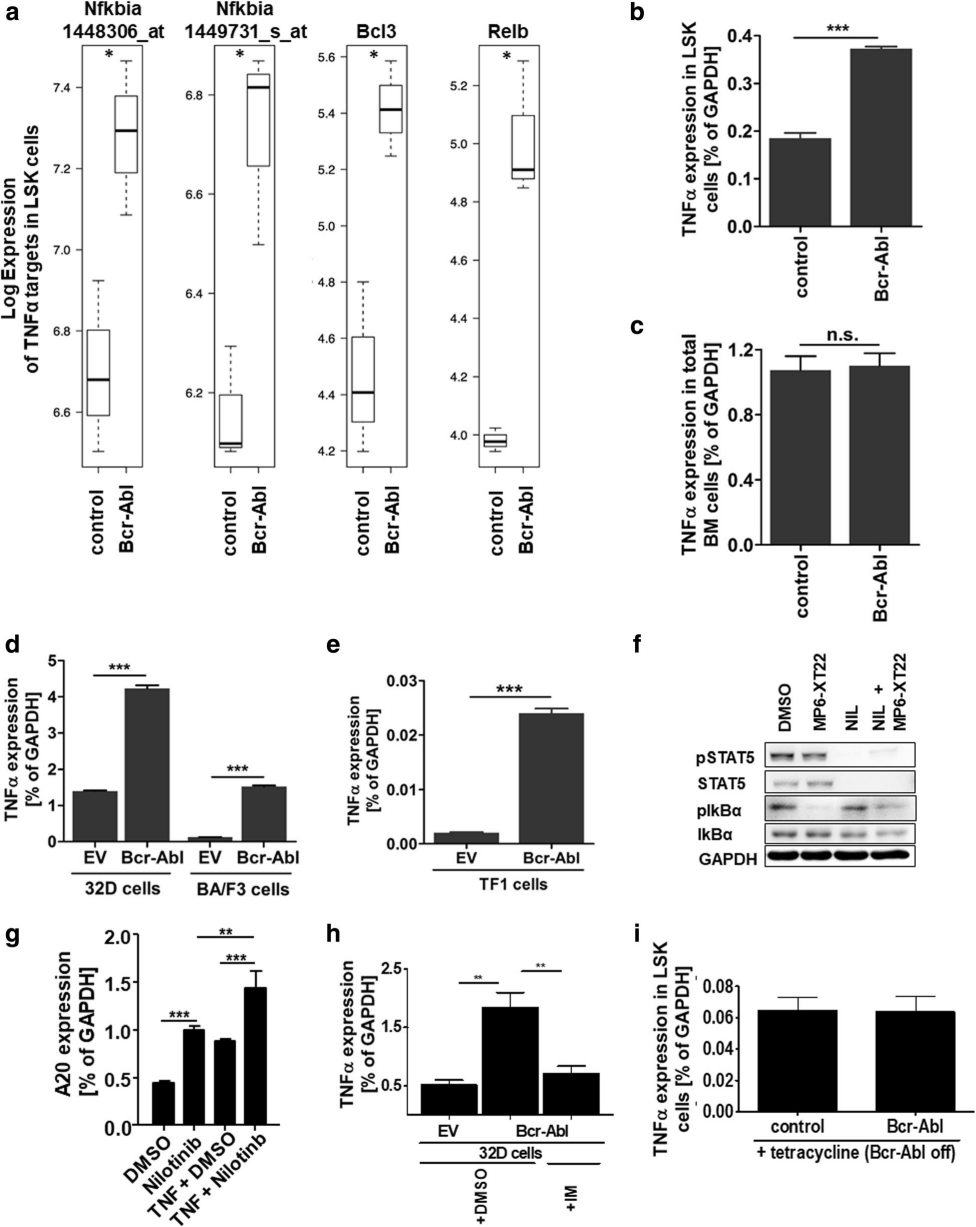
TNFα gene expression signature in leukemic stem and progenitor cells. **a** Gene Set Enrichment Analysis (GSEA) pathway analysis was performed from previously published microarray data (GSE# 18446) that were obtained from SCLtTA/Bcr-Abl and control LSK (lin^−^; Sca-1^+^; c-kit^+^) cells (*n* = 3/3). **b** Expression of TNFα was evaluated in a further set of murine SCLtTA/Bcr-Abl and control LSK cells by qRT-PCR (n = 3/3). **c** qRT-PCR analysis of TNFα in total BM cells from murine SCLtTA/Bcr-Abl and control mice (n = 3/3). **d** TNFα expression was assessed in 32D:EV, 32D:Bcr-Abl, BA/F3:EV and BA/F3:Bcr-Abl cells. **e** Human erythroleukemic cell line TF-1 was transduced with EV and Bcr-Abl and subsequently analyzed for TNFα expression by qRT-PCR. **f** HoxB8 immortalized progenitor BM cells from SCLtTA/Bcr-Abl mice were treated with MP6-XT22 (2 μg/ml), 100 nM nilotinib or the combination of both agents in the presence of 0.05 ng/ml TNFα. Protein expression was analyzed using western blot. **g** Expression of the NF-кB target gene A20 was analyzed in HoxB8 immortalized progenitor BM cells from SCLtTA/Bcr-Abl mice upon Bcr-Abl inhibition using nilotinib (100 nM) in the absence and presence of 0.05 ng/ml TNFα. **h** TNFα expression was analyzed in 32D:Bcr-Abl cells upon imatinib (IM, 5 μM) treatment and **i** in LSK cells from SCLtTA/Bcr-Abl mice that had been induced to express Bcr-Abl for 25 days and were then reverted to not express the oncogene for 48 days. (n.s. = not significant, ^***^p < 0.001)

Using an inducible transgenic CML mouse model (SCLtTA/Bcr-Abl) as well as primary patient material, we previously identified leukemic stem cells (LSC) that persist despite complete Bcr-Abl inhibition [4]. Applying the same mouse model, we here performed Gene Set Enrichment Analysis (GSEA) [31] of our previously published microarray data from CML vs normal LSK (lin^−^; c-kit^+^; Sca-1^+^) cells [24] that are highly enriched for stem cells. These data identified TNFα signaling as the most significant upregulated pathway in CML LSK cells vs controls (Additional file 1). In particular, expression of the NF-κB family members IkBα, BCL-3 and Relb were significantly increased by 1.8-fold using two different IkBα probes as well as 2.5-fold and 2.9-fold respectively (Fig. 1a) reflecting NF-κB activity. Using qRT-PCR, we found that TNFα gene expression was likewise significantly increased in LSK cells from CML mice as compared to controls (2.01-fold, Fig. 1b). Aiming to analyze if TNFα upregulation was specific for the stem and progenitor cell compartment or not, we next tested whole BM cells from control and SCLtTA/Bcr-Abl CML mice in that we induced the disease for 6 days by tetracycline withdrawal which lead to the activation of the oncogenic Bcr-Abl kinase. Analysis of TNFα expression in total BM cells revealed similar levels in control vs leukemic animals (Fig. 1c) showing that TNFα upregulation is specific for the stem and progenitor compartment in the CML mouse model. Next, we analyzed TNFα gene expression in the murine myeloid progenitor cell line 32D and the murine lymphoid progenitor cell line BA/F3 upon Bcr-Abl expression. Both progenitor cell types depend on IL-3 signaling and Bcr-Abl expression induces cytokine-independent growth. TNFα levels increased upon Bcr-Abl expression by 3.02-fold in 32D cells and 12.06-fold in BA/F3 cells (Fig. 1d). In order to test TNFα expression also in human Bcr-Abl positive vs Bcr-Abl negative progenitor cells, we virally infected the human erythroblastic CD34^+^ cell line TF-1 to express BCR-ABL. Again, TNFα expression was highly increased (11.76-fold) in Bcr-Abl positive cells (Fig. 1e).

In order to study if TNFα induced signaling is persisting despite Bcr-Abl inhibition in our model we analyzed phosphorylation of IKBα. Therefore, we first conditionally immortalize early hematopoietic progenitor cell derived from the transgenic SCLtTA/Bcr-Abl model, using an estrogen-regulated HoxB8 variant [27]. IkBα blocks TNFα-induced NF-кB activation by NF-кB binding that prevents its nuclear translocation. Phosphorylation of IKBα induces ubiquitination and thereby degradation of the NF-кB regulating kinase and this allows for NF-кB transcription factor activity. As expected, the presence of the TNFα neutralizing antibody MP6-XT22 abolished IKBα phosphorylation (Fig. 1f). However, nilotinib treatment alone failed to reduce pIKBα and this could be largely overcome by combining MP6-XT22 with nilotinib. TKI persisting TNFα induced NF-кB activation was also shown by expression of A20, as a specific TNFα target gene. Nilotinib treatment did not reduce but rather increased the level of A20 gene expression (Fig. 1g). We proceeded to test if TNFα secretion by the malignant clone is Bcr-Abl dependent. Inhibition of Bcr-Abl using imatinib significantly reduced TNFα expression in 32D Bcr-Abl cells (Fig. 1h) suggesting that in this model TNFα levels depend on the malignant kinase activity. Next, we studied TNFα expression in LSK cells from SCLtTA/Bcr-Abl mice, that were first induced to express Bcr-Abl for 25 days and then reverted to not express Bcr-Abl for 48 days [4]. Expression of TNFα in these previously malignant LSK cells was reverted to normal level (Fig. 1i).

### Pharmacological inhibition of Bcr-Abl and TNFα impairs leukemic progenitor cell growth

**Fig. 2:**
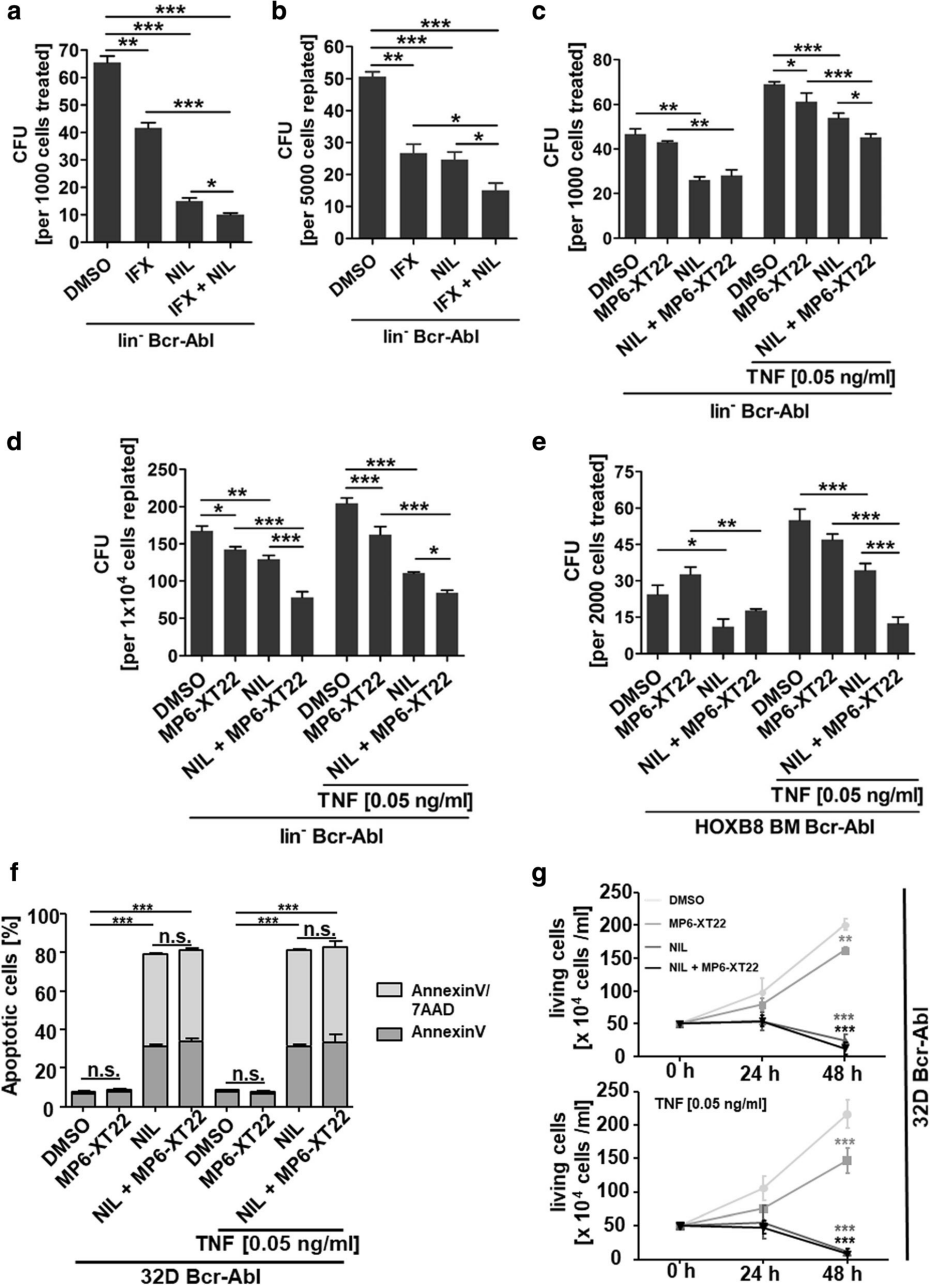
Pharmacological inhibition of Bcr-Abl and TNFα reduces leukemic progenitor cell growth. **a** Lin^−^ SCLtTA/Bcr-Abl BM cells were treated for 72 h with 500 μg/ml IFX, 100 nM nilotinib and the combination of both agents. 1000 treated cells were seeded per ml into methylcellulose. **b** 5000 cells/ml from (a) were used for re-plating. **c** Lin^−^ BM cells from SCLtTA/Bcr-Abl mice were subjected to MP6-XT22 (2 μg/ml), 100 nM nilotinib and the combination of both agents in the absence or in the presence of 0.05 ng/ml TNFα for 72 h. 1000 treated cells/ml cells were seeded into methylcellulose **d** 1 × 10^4^ cells/ml from (c) were used for re-plating. **e** HoxB8 immortalized progenitor BM cells from SCLtTA/Bcr-Abl mice were treated with MP6-XT22 (2 μg/ml), 100 nM nilotinib and the combination of both agents in the absence or presence of 0.05 ng/ml TNFα for 72 h. 2000 treated cells/ml were seeded into methylcellulose. **f** apoptosis was evaluated by AnnexinV/7AAD staining (light grey: Annexin V pos; dark grey: Annexin V/7AAD pos) and **g** proliferation was assessed by cell counting using 32D:Bcr-Abl cells that were subjected to MP6-XT22 (2 μg/ml), 100 nM nilotinib or the combination of both agents, in the absence or in the presence of 0.05 ng/ml TNFα. (n = 3 for each treatment, *p < 0.05, **p < 0.01, ^***^p < 0.001)

As the inflammatory CML microenvironment has previously been shown to support stem cell persistence, we next aimed to evaluate the effect of infliximab (IFX) therapy combined with TKI on the clonogenic potential of CML cells. We first isolated linage negative (lin^−^) BM cells from transgenic SCLtTA/Bcr-Abl mice using magnetic activated cell sorting (MACS) and induced these cells by removal of tetracycline in vitro to express Bcr-Abl. Treatment was performed for 72 h followed by cell seeding into a colony formation (colony forming unit, CFU) assay. Administration of IFX alone already significantly reduced the CFU capacity by 1.6-fold (Fig. 2a). As expected, nilotinib further reduced the CFU capacity by 4.4-fold as compared to control. Interestingly, combinational treatment showed superior effects and diminished CFU capacity by 6.5-fold compared to untreated control. To evaluate self-renewal potential of these cells in vitro, we performed serial plating using 5 × 10^3^ cells per ml without further treatment. Cells that had previously been treated with IFX showed a 1.9-fold decrease in re-plating efficiency and this was similar to nilotinib treated cells showing a 2.1-fold reduction in CFU numbers (Fig. 2b). Combinational treatment was most efficient and reduced clonogenic cells by 3.4-fold. Although IFX has been previously applied to reduce TNFα levels in a variety of mouse models [15–22] a recent report showed that IFX-induced anti-inflammatory response including TNFα reduction in mice is not mediated via direct TNFα binding [23] unlike to human. To further validate our results, we additionally used a murine specific TNFα neutralizing antibody (MP6-XT22, 2 μg/ml) and treated lin^−^ SCLtTA/Bcr-Abl BM cells accordingly but added murine TNFα (0.05 ng/ml) in addition to mimic the increased TNFα levels in patients. CFU capacity was increased (1.5-fold) upon TNFα addition alone in the DMSO control group (Fig. 2c) showing that TNFα indeed supports malignant stem and progenitor cell growth. Moreover, nilotinib treatment was less effective in the presence of added TNFα and reduced CFU potential by only 1.3-fold vs 1.8-fold in the absence of the cytokine showing that TNFα supports the clonogenic potential and reduces the sensitivity to TKI therapy in these cells (Fig. 2c). The effect of the murinized TNFα antibody was less notable as compared to IFX in the first plaiting and could only induce limited but significant reduction of the CFU potential upon replaiting (Fig. 2d). However, cells that had been subjected to the combinational treatment were again significantly reduced in CFU capacity as compared to nilotinib treatment alone (Fig. 2d). Similar results were obtained when we treated Hoxb8 immortalized progenitor cells derived from transgenic SCLtTA/Bcr-Abl mice (Fig. 2e). To study the mechanism resulting in impaired CML cell biology upon TNFα inhibition we applied 32D Bcr-Abl cells that were subjected to TNFα antibody treatment, nilotinib therapy or the combination, upon adding or not adding physiological TNFα concentration. As expected, apoptosis was significantly induced upon nilotinib treatment but not further enhanced upon additional TNFα targeting (Fig. 2f). TNFα targeting alone was not sufficient to induce apoptosis in these CML cells. Using the same model, we subsequently analyzed the effect on proliferation and here we could observe a significant reduction upon TNFα targeting alone. This was evident in CML cells independent of TNFα addition, likely because of TNFα expression per se in these cells (Fig. 1d). Nilotinib treatment by itself largely abolished proliferation in this model and thus there was no additional effect observable (Fig. 2g).

### Anti-inflammatory therapy together with TKI reduces leukemic stem cells in vivo

**Fig. 3:**
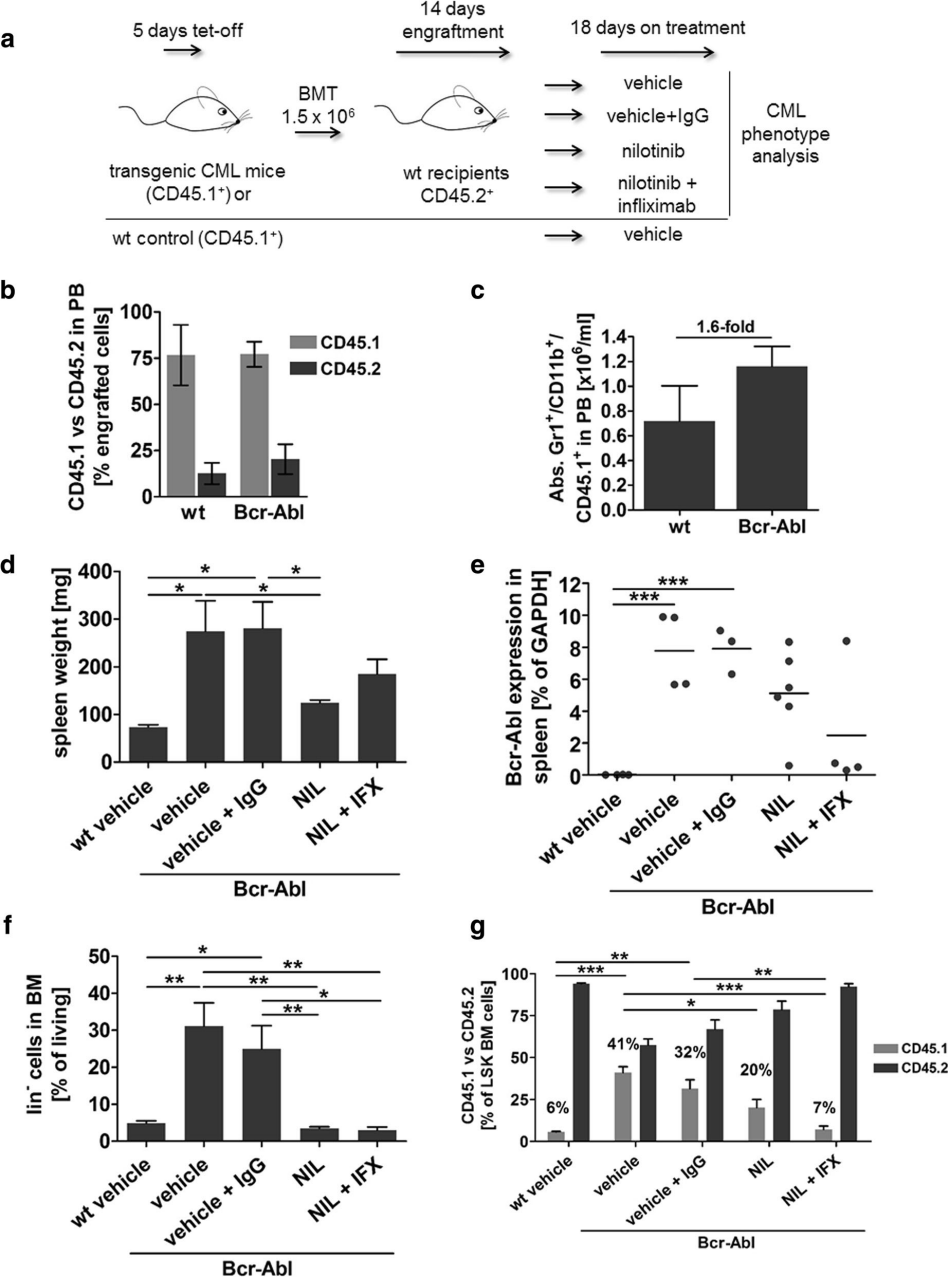
Anti-inflammatory therapy and Bcr-Abl inhibition reduces leukemic stem cells in vivo. **a** Experimental design for the treatment of Bcr-Abl and wt (CD45.1) BM transplanted recipient FVB/N mice (CD45.2). **b** Engraftment of CD45.1^+^ donor cells in the peripheral blood of Bcr-Abl and wt transplanted mice was evaluated by FACS 14 d after BMT (n = 3/3). **c** Absolute Gr-1^+^/ CD11b^+^/ CD45.1^+^ cell number of wt and Bcr-Abl transplanted recipients in peripheral blood 14 d after BMT (n = 3/3). **d** Upon autopsy spleen weight of FVB/N wt and FVB/N Bcr-Abl transplanted recipients with the indicated treatment was determined. **e** Bcr-Abl mRNA expression was analyzed in spleen cells of recipient mice by qRT-PCR. **f** FACS analysis of lin^−^ cell population of FVB/N wt and FVB/N Bcr-Abl transplanted recipients which received the indicated therapy. **g** Distribution of donor (CD45.1) and recipient (CD45.2) derived cells within the LSK (lin^−^, c-kit^+^, Sca-1^+^) cell compartment of transplanted mice with the indicated treatments. (*n* = 4/ 4/ 3/ 6/ 4, *p < 0.05, **p < 0.01, ^***^p < 0.001)

To evaluate the effect of combined anti-inflammatory and TKI treatment on CML stem cells in vivo, we next tested the effect of nilotinib together with infliximab treatment in CML mice. To allow for discrimination of donor vs recipient cells we transplanted 1.5 × 10^6^ CD45.1^+^ SCLtTA/Bcr-Abl BM cells into congenic FVB/N CD45.2^+^ recipients (Fig. 3a). Donor mice were induced to express Bcr-Abl by tetracycline withdrawal from the drinking water provoking splenomegaly in Bcr-Abl positive mice (data not shown). Total BM cells from Bcr-Abl positive mice were isolated and transplanted into 10 Gy irradiated recipients. Two weeks after transplantation peripheral blood (PB) analysis showed similar engraftment of transplanted cells in both recipient groups (Fig. 3b) and revealed a trend of increased (1.6-fold) donor-derived CD45.1^+^; Gr-1^+^; CD11b^+^ neutrophils in recipients of Bcr-Abl positive BM cells as expected (Fig. 3c). Subsequently, Bcr-Abl positive control mice were treated with vehicle or vehicle combined with human IgG (10 mg/kg weekly) to control for the TKI solvent as well as the chimeric antibody IFX (*n* = 6 per group). Further, 2 Bcr-Abl positive cohorts were treated either with nilotinib (50 mg/kg daily) or the combination of nilotinib and IFX (n = 6 each group). Recipients of Bcr-Abl negative wt cells were implemented to control for disease development and were treated with vehicle. The therapy was continued for 18 days whereas several mice died during that period (2 Bcr-Abl mice treated with vehicle, 3 Bcr-Abl mice treated with vehicle and IgG as well as 2 Bcr-Abl mice treated with nilotinib and IFX). Neither of the 2 mice treated with nilotinib and IFX showed a splenomegaly at that stage whereas most of the vehicle or vehicle and IgG mice did (Table 1). Remaining mice were analyzed 18 days after therapy start and spleen weight was determined in all recipients (Fig. 3d). Mice that had been transplanted with Bcr-Abl positive BM cells and were treated with control agents developed splenomegaly as compared to wt. Nilotinib alone significantly reduced spleen weight by 2.20-fold and a trend in reduction was likewise observed in mice that received IFX and nilotinib (1.52-fold) compared to vehicle and IgG control. Analysis of spleen cells confirmed Bcr-Abl mRNA expression in vehicle, vehicle and IgG, nilotinib and nilotinib and IFX treated animals (Fig. 3e). While nilotinib treatment could only induce a mild reduction in Bcr-Abl expression levels upon 18 days of treatment, the combination of nilotinib and IFX further reduced Bcr-Abl mRNA levels. Next we analyzed lin^−^ BM cells. Transplantation of Bcr-Abl positive BM increased lin^−^ cells in animals treated with control substances as expected (Fig. 3f) and TKI as well as combined treatment significantly reduced this cell fraction by 9.06-fold and 8.22-fold respectively. As nilotinib alone was extremely effective in reducing lin^−^ BM cells, we could not observe an additional effect by adding IFX therapy at the progenitor cell level. We then analyzed BM LSK^+^ cells (lin^−^; c-kit^+^; Sca-1^+^) in these mice. Upon transplantation of wt BM we identified 6% (±0.8) donor derived non leukemic CD45.1^+^ cells within the LSK compartment defining the baseline for non-leukemic LSK engraftment and expansion in this setting (Fig. 3g). Bcr-Abl positive mice, treated with vehicle or vehicle and IgG showed a significant increase in leukemic donor-derived CD45.1^+^ LSK cells (vehicle 41% (±6.9); vehicle + IgG 32% (±12.8) that did not differ significantly between both control groups. As expected nilotinib treatment significantly decreased leukemic LSK cells by 2.03-fold compared to control treated mice. Interestingly, we observed a 4.38-fold reduction in leukemic LSK cells upon combined nilotinib and IFX treatment showing that the combination is capable of further diminishing TKI induced reduction of leukemic LSK cells by 2.81-fold (Fig. 3g). Taken together, these data show that combined treatment significantly enhances decline of malignant stem cells and reduces the level of these cells down to 7% (±3.9) that is marginally above wt baseline of 6%.

**Table 1:** Mortality during treatment

Transplanted cells	Treatment	Day of treatment (spleen size)
1.5 × 10^6^ Bcr-Abl BM	vehicle	day 8 (380 mg), day 12 (n.a.)
1.5 × 10^6^ Bcr-Abl BM	vehicle + IgG	day 9 (274 mg), day 12 (410 mg), day 13 (77 mg)
1.5 × 10^6^ Bcr-Abl BM	nilotinib	none
1.5 × 10^6^ Bcr-Abl BM	nilotinib + infliximab	day 7 (91 mg), day 9 (108 mg)
1.5 × 10^6^ wt BM	vehicle	none

### Serial transplantation reveals impaired malignant stem cell function in mice receiving combination therapy

**Fig. 4:**
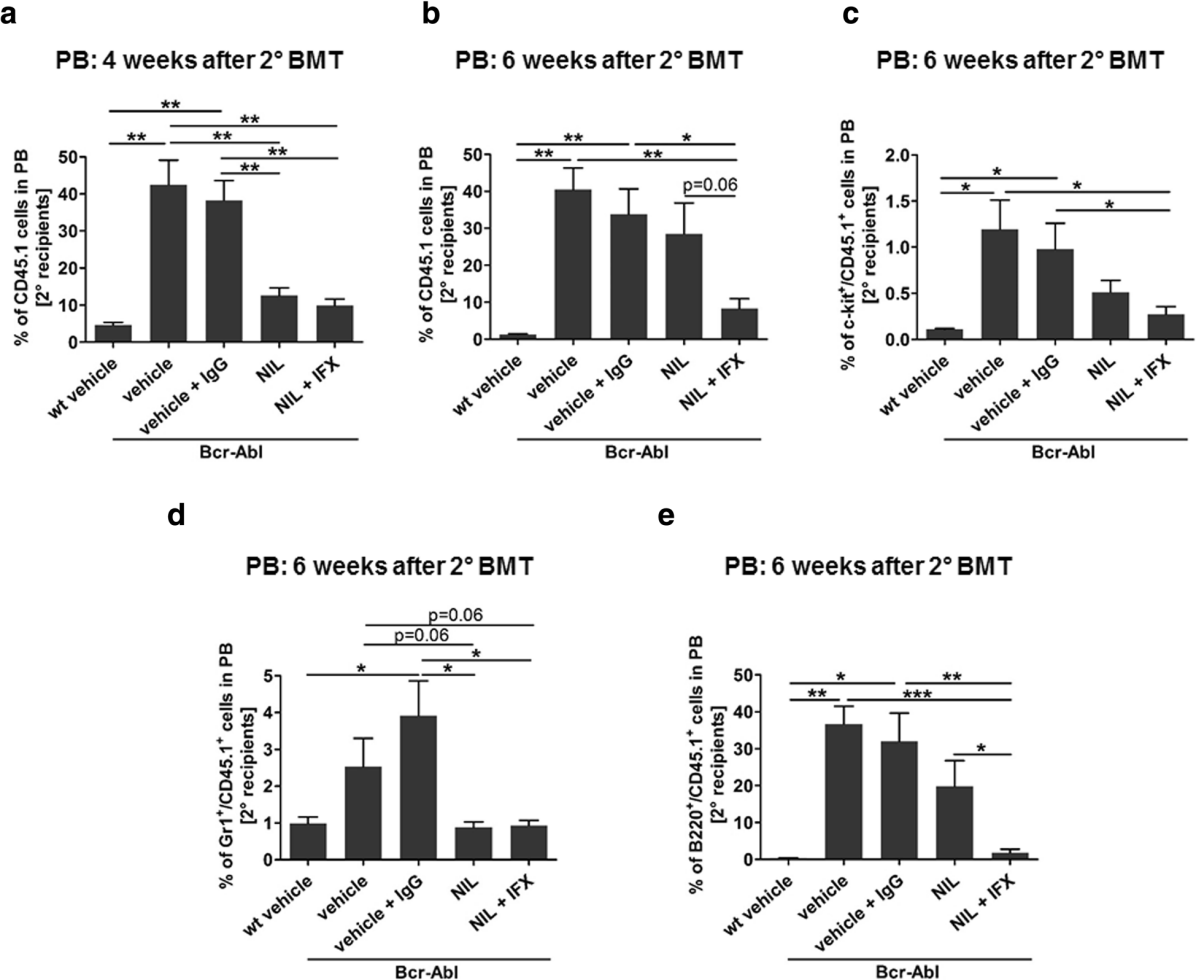
Serial transplantation reveals impaired malignant stem cell function in mice receiving combination therapy. 3.5 × 10^6^ CD45.1^+^ BM cells from FVB/N wt recipients treated with vehicle and FVB/N Bcr-Abl recipients treated with vehicle, vehicle + IgG, NIL, and NIL+ IFX were transplanted into irradiated secondary CD45.2 FVB/N recipients. **a** Peripheral blood (PB) from secondary recipients for donor derived CD45.1^+^ cells was assessed by FACS 4 weeks after BMT. 6 weeks after 2nd BMT PB was analyzed by FACS for **b** donor derived CD45.1^+^, **c** c-kit^+^/CD45.1^+^, **d** Gr-1^+^/CD45.1^+^, and **e** B220^+^/CD45.1^+^ cells. (*n* = 3/4/4/4/4, *p < 0.05, **p < 0.01, ***p < 0.001)

Aiming to test the LSC quality after treatment, we next performed serial transplantation experiments. 3 × 10^6^ leukemic CD45.1^+^ BM derived cells from vehicle, vehicle + IgG, nilotinib, and nilotinib + IFX treated animals as well as wt controls were re-transplanted into irradiated congenic FVB/N recipients (CD45.2^+^). We applied no further treatment in these secondary recipients and monitored the survival as well as the capacity of transplanted cells to induce early malignant cell expansion. Survival was significantly impaired in vehicle and vehicle + IgG re-transplanted mice as compared to nilotinib and nilotinib + IFX treated cell recipients (Additional file 2). No difference was observed in secondary recipients receiving either nilotinib or nilotinib + IFX treated cells presumably due to transplantation of high cell numbers that impair resolution of this endpoint. However, PB analysis showed clear differences in initial malignant cell expansion. Evaluation 4 weeks after re-transplantation revealed a significant engraftment of CD45.1^+^ donor derived cells in Bcr-Abl positive vehicle and vehicle + IgG re-transplanted animals (Fig. 4a). As expected a significant reduction of CD45.1^+^ donor derived cells was detected in nilotinib (3.36-fold) and nilotinib + IFX (3.89-fold) re-transplanted mice compared to vehicle and vehicle + IgG controls confirming that both treatments affected malignant stem and progenitor cells (Fig. 4a). Interestingly 6 weeks after re-transplantation, analysis of PB showed that leukemic CD45.1^+^ cells of nilotinib re-transplanted animals recovered and increased while this was not the case in cell recipients of double treated mice at that time (Fig. 4b). Further analyses of the PB revealed that donor-derived blasts (CD45.1^+^; c-kit^+^) were increased in recipients of Bcr-Abl positive BM and there was a trend of less CD45.1^+^; c-kit^+^ cells being present in nilotinib re-transplanted mice that was further continued and reduced by 1.88-fold upon combinatory treatment (Fig. 4c). Donor neutrophils (Gr1^+^; CD45.1^+^) were not increased in the nilotinib re-transplanted animals compared to re-transplanted nilotinib + IFX mice (Fig. 4d). Instead, the donor-derived re-expanding cells were positive for the B-cell marker B220 (Fig. 4e) and there was a significant reduction in these cells due to the combinatory treatment as compared to nilotinib monotherapy (11.22-fold). Taken together these data suggest that reduction of FACS positive malignant stem cells seen upon combined treatment in the primary recipients translates into a reduced tendency of these cells to re-expand upon serial transplantation. However, additional experiments would be required to fully address the effect on LSC function.

## Discussion

TKI therapy eliminates the mature leukemic clone in the majority of CML patients but curing the disease by tackling LSC still requires a deeper understanding of the mechanisms allowing for persistence.

A recent report achieved to analyze Bcr-Abl positive vs Bcr-Abl negative stem cells using single-cell RNA sequencing technology. In this article, the authors were able to dissect two distinct LSC subpopulations that are characterized by either a proliferative or a quiescent expression profile [14]. Quiescent LSC expanded during treatment and were associated with inflammatory signatures including TGFβ und TNFα signaling.

By GSEA analysis we here show that TNFα signaling is the most significant upregulated pathway within the LSK compartment of transgenic CML mice. In these mice TNFα has previously been shown to be elevated in plasma, BM and spleen along with further cytokines including MIP-1 α, MIP-1β, G-CSF, IL-1 α, IL-1 α and IL-6 [32]. Addition of TNFα, MIP-1α and MIP-1β selectively increased CML LT-HSC expansion in vitro in this study. Significant elevation of TNFα in CML patients has likewise been reported upon diagnosis and interestingly this remained at high levels even after 6 months of TKI therapy [9]. Moreover, an autocrine TNFα loop in human CML stem cells has already been shown to persist besides Bcr-Abl kinase inhibition in vitro [33] implying that this could present an attractive stem cell specific target. Indeed, inhibition of TNFα by a small molecule induced apoptosis in CML stem and progenitor cells in vitro [33]*.* In line with these data, our own data show that addition of TNFα enhanced CFU capacity upon first and serial plating in primary murine CML cells confirming that the cytokine preserves malignant stem cell quality. Additionally, we observed impaired serial plaiting efficiency upon human (IFX) or murine (MP6-XT22) TNFα antibody treatment combined with nilotinib validating that stem cell quality is impaired due to this therapeutic approach. Although we clearly show elevated TNFα expression in Bcr-Abl positive cells, our data also suggest that this is dependent on the malignant kinase, at least in a murine myeloid progenitor cell line. Likewise, we observed reduction of TNFα expression in LSK cells upon long-term reversion of Bcr-Abl expression. However, this could also be due to re-expansion of Bcr-Abl negative LSK cells upon inhibition of the kinase as we have studied the expression of TNFα 48 days after Bcr-Abl reversion in this model. In primitive human LSCs, TKI persistent TNFα expression has been demonstrated [14, 33]. Yet, additional cell populations could contribute to elevated TNFα levels that are observed in CML mice and patients. This also ties in with the recent finding that CML-derived osteoblasts show elevated levels of TNFα expression, in the SCLtTA/Bcr-Abl model [34].

In another MPN entity an autocrine TNFα function was previously described to support malignant stem cell expansion. Addition of TNFα to human CD34^+^ cells increased cell growth in JAK2V617F positive stem cells [35]. Moreover, TNFα was required for expansion of JAK2V617F cells in a murine transplantation model [36] implying that the LSC promoting TNFα function could be a general phenomenon in MPNs.

Studying the effect of TNFα antibody treatment using our murine primary lin^−^ CML cells revealed a stronger effect on CFU reduction by IFX as compared to the MP6-XT22 antibody. This observation could be explained by a recent report showing that IFX induces its effects in mice independent of direct TNFα binding [23], although reduction of TNFα upon IFX treatment has been documented in various mouse models [15–20]. The mechanism of IFX induced reduction of murine TNFα is unclear. It is speculated that the human IgG part of the chimeric antibody might induce apoptosis in TNFα secreting cells. However, at this stage it cannot be excluded that IFX-induced effects, independent of TNFα, could contribute to the response of CML cells observed in this study. Upon serial transplantation, we observed a non-significant 1.88-fold reduction in donor-derived c-kit^+^ cells and a significant 11.22-fold reduction in donor-derived B220^+^ cells due to combined IFX and nilotinib treatment as compared to nilotinib treatment alone. While the reduction in blasts can be assigned to reduced CML disease the mechanism inducing B-cell reduction is unclear at present but it has been discussed that IFX can alter B-cell biology in treated patients [37, 38] suggesting that this could rather reflect an effect of the antibody treatment itself.

Besides the reduction of TNFα additional inflammatory cytokines such as INFγ, IL-10 [18] and IL-6 [19] were shown to be reduced in IFX treated mice. We previously demonstrated that the spleen is a reservoir for potent LSC in the SCLtTA/Bcr-Abl mouse model [39] and we analyzed expression of inflammatory cytokines in the spleen of treated mice. While IL-10 and IL-6 were not changed by IFX treatment (data not shown) we found INFγ expression to be affected: INFγ was downregulated upon CML development and this was partially reverted upon nilotinib treatment while the combination of nilotinib and IFX again antagonized this effect and decreased IFNγ expression level (Additional file 3). Intriguingly, IFNγ has previously been shown to increase CML CD34^+^ CFU numbers [40] and reduce TKI-sensitivity of CML cells in vitro [41]. Moreover, therapeutic infusion of cytotoxic T cells (CTL) expanded the LSC compartment in a murine model of late stage CML and this was permitted via IFNγ secretion of these CTL [40]. A further report showed that IFNγ induces BCL6 expression in CML cells [42] and BCL6 has already been shown to be critical for LSC survival [43]. As combined nilotinib and IFX therapy reduced IFNγ expression this could potentially allow for a more potent TKI effect on the LSCs in our model. The mechanism inducing reduced IFNγ expression is unclear at present. However, it has been shown that IFX impairs the frequency of IFNγ-secreting cells. Natural killer cells in rheumatoid arthritis patients were reduced upon IFX therapy [44] and in ulcerative colitis patients derived cells, IFX treatment decreased the proliferation of CD4^+^ and CD8^+^ T-cells as well as their secretion level of IFNγ and TNFα, among other cytokines [45]. We have not studied the IFX effect on NK or T-cell populations in the SCLtTA/Bcr-Abl model, yet these data tempt to speculate that IFX-mediated activity on NK or T-cell subsets could also be involved in the pathophysiological effects observed in our study.

As a pleiotropic cytokine, TNFα is involved in pro- as well as anti-inflammatory processes and immunosuppressive mechanisms. In this regard, TNFα has been shown to impair conventional T cell survival [46] or promote immunosuppressive cells, such as myeloid-derived suppressor cells (MDSC) [47, 48] or regulatory T cells (Tregs) [49, 50]. Along this line, IFX therapy in sarcoidosis patients has been shown to reduce elevated frequency of Tregs [51]. Interestingly, CML patients show elevated levels of MDSCs [52, 53] and Tregs [54]. Moreover, CML derived MDSCs themselves have been suggested as a source of TNFα [55], tempting to speculate that TNFα inhibition could also impact on CML biology, not only by direct effects on the malignant stem cell itself but also by supporting a tumor promoting niche.

In several solid cancer entities, TNFα contributes to a pro-carcinogenic microenvironment by activation of NF-κB signaling that promotes cell survival [56, 57]. IFX therapy in mice significantly reduces phosphorylation of RelA (p65) that is a member of the NF-κB transcription factor family [21]. Inhibition of NF-кB signaling via overexpression of a superrepressor mutant of inhibitory IKBα protein has been shown to impair leukemogenesis in a retroviral model of Bcr-Abl driven disease [58]. In an AML mouse model of Bcr-Abl and NUP98-HoxA9 induced disease autocrine TNFα secretion permitted NF-кB activation in LSC and expanded this disease-initiating cell population [59]. These data tempt to speculate that TNFα could also be involved in advanced CML.

## Conclusion

TNFα signaling is induced in CML stem cells and anti-inflammatory therapy elevates TKI induced clonogenic growth reduction. Compatible with this, anti-inflammatory therapy in CML mice enhances TKI induced decline of LSK-cells confirming that successful targeting of CML stem and progenitor cells can be enhanced via addressing their malignant microenvironment simultaneously.

## Additional files

Additional file 1: Gene Set Enrichment Analysis of LSK microarray data from CML mice (SCLtTA/Bcr-Abl) vs controls (SCLtTA and wt). (XLSX 19 kb)

Additional file 2: Combined NIL + IFX therapy does not prolong survival in 2nd recipients. 3.5 × 10^6^ CD45.1^+^ BM cells from FBV/N wt recipients treated with vehicle and FVB/N Bcr-Abl recipients received vehicle, vehicle + IgG, NIL, and NIL + IFX were transplanted into irradiated secondary CD45.2 FVB/N recipients. Survival was monitored for 150 days (*n* = 3 for wt recipients, *n* = 4 for Bcr-Abl recipients, **p < 0.01). (TIF 142 kb)

Additional file 3: INF-γ expression is altered due to NIL and IFX in Bcr-Abl transplanted mice. mRNA level of INF-γ was analyzed by qRT-PCR in spleen cells of FVB/N wt and FVB/N Bcr-Abl transplanted recipients with the indicated treatment. Values were normalized to wt vehicle control (n = 3 for wt, n = 4 for vehicle, *n* = 6 for vehicle + IgG and NIL, *n* = 5 for NIL + IFX; *p < 0.05, **p < 0.01) (TIF 172 kb)

## Data Availability

The dataset analyzed in this study is available under GSE18446; https://www.ncbi.nlm.nih.gov/geo/query/acc.cgi?acc=GSE18446. All additional data generated and analyzed are included in this published article and its supplementary information files.

## Abbreviations

BM: Bone marrow
CFU: Colony forming unit
CML: Chronic myeloid leukemia
CP: Chronic phase
IFX: Infliximab
LSC: Leukemic stem cell
MPN: Myeloproliferative neoplasm
PB: Peripheral blood
TKI: Tyrosine Kinase Inhibitor
TNF: Tumor necrosis factor
wt: Wildtype
