# Supplementary figures and images for: Infliximab therapy together with tyrosine kinase inhibition targets leukemic stem cells in chronic myeloid leukemia
Source: BMC Cancer. 2019 Jul 4;19:658. doi: 10.1186/s12885-019-5871-2 (PMC6610865; doi:10.1186/s12885-019-5871-2)

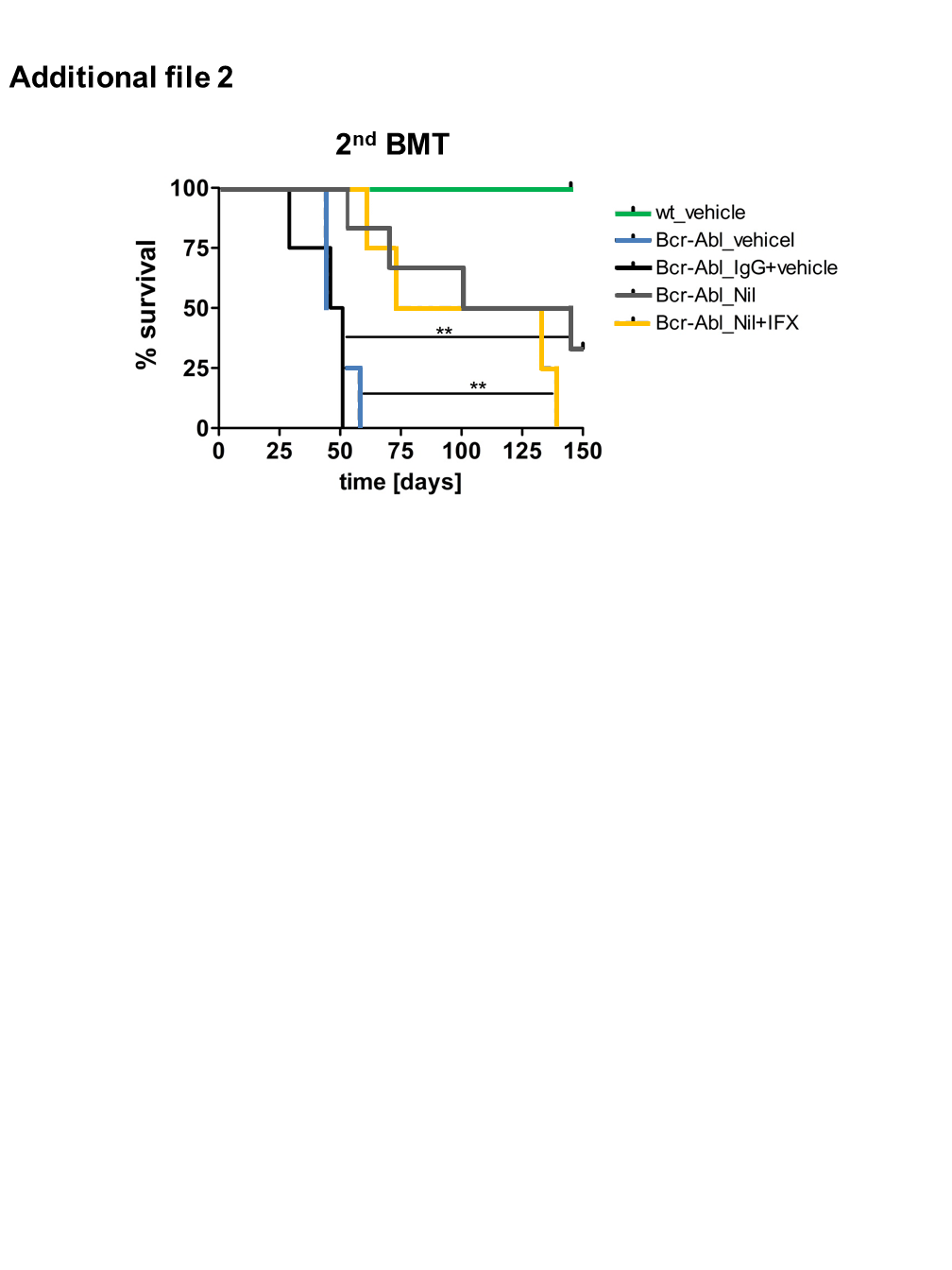

Supplement: Supplementary file 2 — Combined NIL + IFX therapy does not prolong survival in 2nd recipients. 3.5 × 10^6^ CD45.1+ BM cells from FBV/N wt recipients treated with vehicle and FVB/N Bcr-Abl recipients received vehicle, vehicle + IgG, NIL, and NIL + IFX were transplanted into irradiated secondary CD45.2 FVB/N recipients. Survival was monitored for 150 days (n = 3 for wt recipients, n = 4 for Bcr-Abl recipients, **p < 0.01). (TIF 142 kb) [file 12885_2019_5871_MOESM2_ESM.tif]

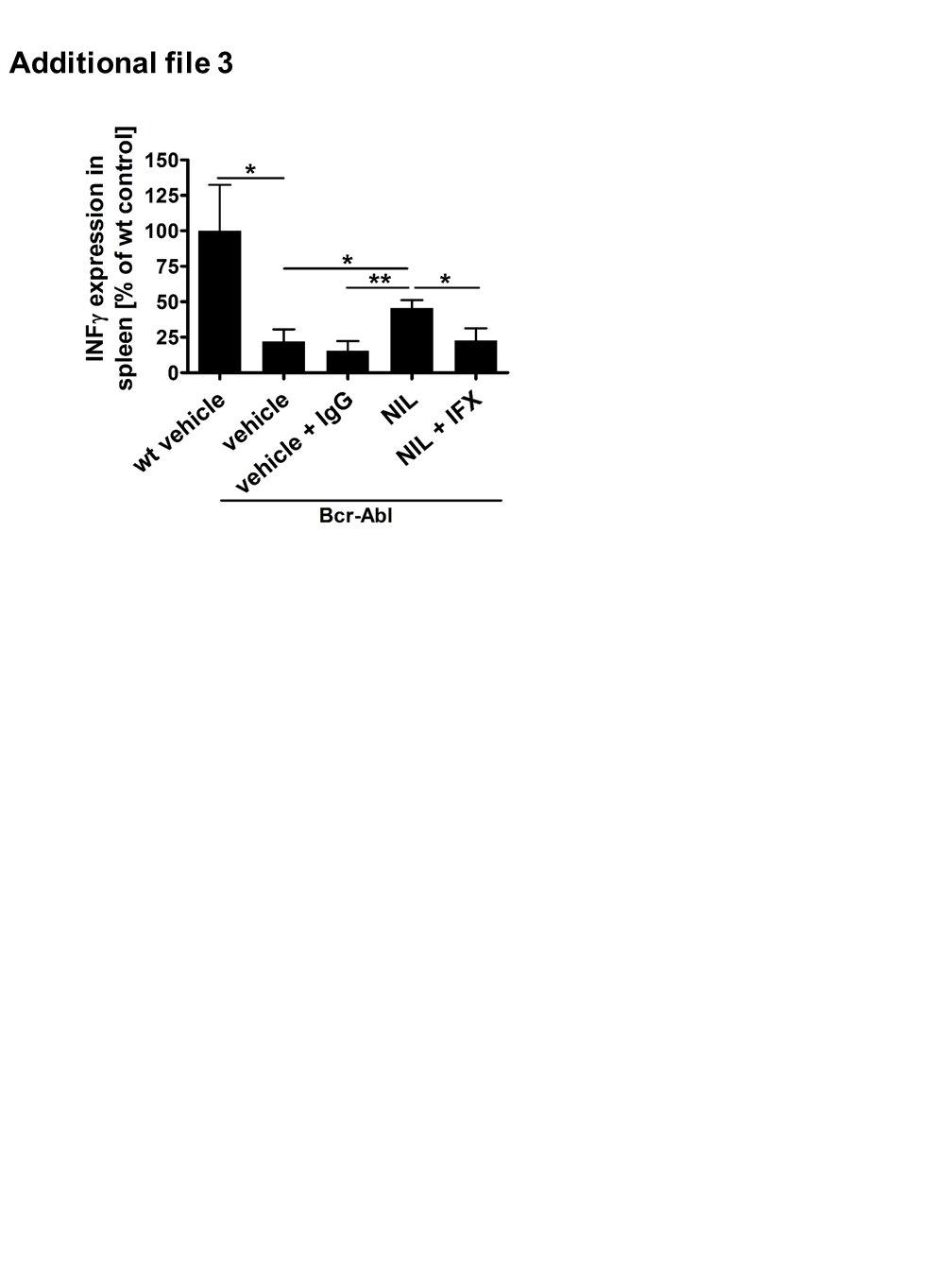

Supplement: Supplementary file 3 — INF-γ expression is altered due to NIL and IFX in Bcr-Abl transplanted mice. mRNA level of INF-γ was analyzed by qRT-PCR in spleen cells of FVB/N wt and FVB/N Bcr-Abl transplanted recipients with the indicated treatment. Values were normalized to wt vehicle control (n = 3 for wt, n = 4 for vehicle, n = 6 for vehicle + IgG and NIL, n = 5 for NIL + IFX; *p < 0.05, **p < 0.01) (TIF 172 kb) [file 12885_2019_5871_MOESM3_ESM.tif]
